# Use of Weight Loss Supplements According to the Purported Mechanisms of Action Among Polish Adults Living in an Obesogenic Environment: The Role of Sociodemographic and Health-Related Factors

**DOI:** 10.3390/nu17243963

**Published:** 2025-12-18

**Authors:** Paulina M. Nowaczyk, Krzysztof Durkalec-Michalski, Adrian Lubowiecki-Vikuk, Adam Kantanista

**Affiliations:** 1 Department of Sports Dietetics, Poznan University of Physical Education, 61-871 Poznan, Poland; durkalec-michalski@awf.poznan.pl; 2 Polish Society of Nutritional Sciences, 02-776 Warsaw, Poland; 3 Institute of Management, SGH Warsaw School of Economics, 02-554 Warsaw, Poland; alubow@sgh.waw.pl; 4 Department of Physical Education and Lifelong Sports, Poznan University of Physical Education, 61-871 Poznan, Poland; kantanista@awf.poznan.pl

**Keywords:** weight loss supplements, overweight, obesity, thermogenesis, appetite suppressants, anthropometry, physical activity

## Abstract

**Background/Objectives**: Weight loss supplements (WLS) are popular dietary agents; however, their effectiveness is rarely supported by scientific-based evidence. This cross-sectional study aimed to identify the types of WLS used among adult Poles in relation to sociodemographic factors, anthropometric indices, and physical activity (PA). **Methods**: The study was conducted among 1071 adults aged 19–80 years in Świętochłowice, a region in Poland with a high rate of overweightness and obesity. Body mass index (BMI), waist-to-hips ratio, and weight-to-height ratio were calculated. Fat mass percentage was assessed. Data on PA level and the use of WLS were collected via a face-to-face questionnaire. **Results**: Nearly 70% respondents declared consumption of WLS, including 486 females and 259 males. Among WLS consumers, 43.4% had normal BMI, and 6.3% were underweight. The most popular WLS were supplements aiming at enhancing thermogenesis (38.0% of WLS consumers), followed by WLS decreasing appetite (15.0%) and blocking dietary fat absorption (13.0%). Sociodemographic factors (sex, age, marital status, education and financial status) were strong determinants of using particular types of WLS. Mean values of anthropometric indices and PA level were different among consumers of various WLS. **Conclusions**: WLS use was common, including among individuals without overweightness/obesity, or cardiometabolic risk. Extensive educational programs and legislation are essential to promote justified, rational, effective, and safe methods for reducing excessive body mass.

## 1. Introduction

Weight loss requires negative energy balance, what can be achieved via reduction in energy intake, or increase in energy expenditure (by increase in physical activity and/or thermogenesis), or both conditions together. Weight loss management strategy should ensure a 0.5–1 kg reduction in body mass (BM) per week. It was previously suggested [1] that moderate BM loss (5–10% reduction in BM) leads to clinically meaningful improvements in numerous health outcomes. According to the recent systematic review by Dhar et al. [1], even low BM loss level (<5% of BM) can lead to improvements in cardiovascular, metabolic, renal and hepatic, inflammatory, ovulatory and psychosocial measures that are likely to result in health improvements. Still, the long-term effectiveness of dietary BM loss programs is limited [2]. In the study by Purcell et al. [2], it was shown that regardless of BM loss strategy (rapid vs. gradual BM loss), 12 weeks of BM reduction resulted in a 14.3–14.6 kg reduction; however, after 144 weeks of follow-up, it was found that the reduction was scarcely about 4.1–4.3 kg. Thus, the percentage of BM regain ranged from 70.5 to 71.2%.

The finding by Purcell et al. [2] indicated that a 15% BM loss can be achieved in about 80% of individuals without the use of pharmacotherapy (i.e., drugs, supplements). According to the recommendations on dietary treatment of obesity in adults [3], incorporation of pharmacotherapy during weight loss programs can be considered in individuals experiencing the second (BMI 35–39.9 kg/m^2^) or third (BMI ≥ 40.0 kg/m^2^) degree of obesity. Moreover, during BM loss ingestion of WLS should not be routinely recommended. Nevertheless, the dietitian may consider enriching the nutritional treatment process through targeted and individualized use of WLS. In addition, scientific research must provide evidence-based supplementation background and confirm the safety and effectiveness of implemented treatment [3].

The more frequent use of non-prescription WLS seems to be common among individuals who struggle to maintain an optimal body weight. It was estimated that 15.2% of American adults have, at some point, used WLS, with the highest use among females aged 18 to 34 years [4]. Moreover, we previously observed an alarming percentage of 65.9% of adults using WLS [5]. Among Polish adults aged 18–30 (42% of participants), 31–45 (41%), and 46–60 (34%) years, the most used source of information on dietary supplements is the Internet, followed by family; while in adults aged > 60 years it is family at the forefront (33% of participants) followed by the Internet (25%) [6]. Based on the analysis by Ng et al. [7] the quality of online information on dietary and herbal supplements for BM loss is diverse. The highest-quality information was provided by websites categorized as health portals and government websites, while the lowest-quality information websites were commercial ones. Thus, healthcare providers need to be actively aware of this and need to guide patients for high-quality WLS information sources and to support safe, effective, and evidence-based decisions relating to WLS use.

It should also be underlined that the safety of dietary supplements is often questionable [8]. For instance, in 2021 in Poland, ethylene oxide had already been detected in meat, cold cuts, ice cream, spices, and food supplements. Alert notifications were reported for WLS aids containing plant extracts (e.g., bamboo shoot extract), appetite suppressants, or vitamin preparations. The Chief Sanitary Inspectorate in Poland regularly announces the withdrawal of certain batches of food supplements due to the use of an ingredient contaminated with ethylene oxide [9]. It must also be mentioned that the great majority of agents advertised as weight loss enhancers available on Polish market are classified as dietary supplements. From a legal standpoint within the European Union, dietary supplements are classified as food products under Directive 2002/46/EC [10] and Regulation (EC) No 178/2002 [11] and, as such, are regulated by food-law provisions and may not be presented as medicinal products (i.e., they cannot claim therapeutic or pharmacological effects). On the other hand, pharmaceuticals (also known as medicinal products) are regulated under Directive 2001/83/EC [12] and require formal marketing authorization based on demonstrated pharmacological, immunological, or metabolic efficacy. Examples of weight loss agents available on Polish and European market, that can be (still not always are) categorized as pharmaceuticals, are products containing glucomannan. However, each time, the label should be carefully checked to verify whether a given product is a dietary supplement or a pharmaceutical.

The high number of people using supplements is of additional concern, as consumption trends to be among overweight or obese people. The latter are likely to be taking drugs, which, when combined with WLS, increases the risk of interactions and can lead to health disturbances [13]. Therefore, the aim of this cross-sectional study was to identify the WLS (classified according to their purported mechanism of action) use among adult Poles from towns with high obesity rates in relation to sociodemographic factors, anthropometric parameters, and physical activity.

## 2. Materials and Methods

### 2.1. Study Participants

The cross-sectional study included 1071 participants aged 19–80 years (45.4 ± 15.7 years). The survey was conducted among adult residents of one of the three provinces in Poland, i.e., the Silesian Voivodeship, in which there is a high percentage of overweight and obese people [14]. The residents of the town of Świętochłowice were arbitrarily selected, due to the characteristics typical of an obesogenic environment [15]. First, Świętochłowice is one of the most densely populated towns in Poland and Europe (there are more than 3785 people per 1 km^2^) [16]. Second, the behavior of its residents is typical of the sedentary lifestyle of the region’s communities, and their eating habits correspond to the traditional Silesian way of eating, based on high-energy foods [17,18,19]. Still, residents are not homogeneous and may share different values regarding obesity in their place of residence [20]. Convenience sampling was implemented, as it is the most common strategy in developmental sciences [21]. Convenience sampling is also cost-effective, efficient, and simple to implement [22], and enabled us to carry out the current cross-sectional study.

### 2.2. Evaluation of WLS Use

The use of WLS was evaluated based on an author-designed questionnaire. Firstly, participants were asked whether they had used any WLS in the previous 3 months (‘yes’ or ‘no’ answers were possible). If the ‘yes’ answer was given, respondents were asked to list the trade name of supplements they had used. Next, it was decided whether the given product was a WLS, and if ‘yes’, the product was classified into one of the following categories of WLS according to its purported mechanism of action: (1) *enhancing thermogenesis* (e.g., including L-carnitine, hydroxycitric acid, green tea, caffeine, licorice, bitter orange, guarana, yerba mate, conjugated linoleic acid); (2) *decreasing appetite* (e.g., guar gum, pineapple, glucomannan, psyllium, and other fibers); (3) *blocking dietary fat absorption* (e.g., chitosan); (4) *increasing water elimination* (e.g., stinging nettle, birch); (5) *enhancing biochemical metabolism* (e.g., chromium, ginseng); (6) *regulating metabolic rate*; and (7) *exhibiting complex/miscellaneous mechanisms* [23,24,25]. Many dietary supplements, including WLS, contain multiple active compounds whose mechanisms of action overlap or are unclear based on the literature [23]; thus, for the purpose of the investigation, it was decided to divide WLS according to their ’purported‘ mechanism of action. The categorization considered the main active compound declared by the manufacturer. For multi-ingredient preparations and/or if it was impossible to identify a single mechanism of action, the group “*exhibiting complex/miscellaneous mechanisms*” was included in the analysis. The questionnaires were filled with the supervision of interviewers with a degree in Pharmacy.

### 2.3. Anthropometric, Body Fat and Physical Activity Level Evaluation

Body mass was measured using an Omron scale (Omron Healthcare, Inc., Vernon Hills, IL, USA) in light clothing (without shoes) to the nearest 0.5 kg. Height was measured with an anthropometer Seca 213 (Seca Gmbh & Co., Hamburg, Germany) to the nearest 0.5 cm. Body mass index was calculated and the following classification was applied—underweight: <18.50 kg/m^2^; normal body mass: 18.50–24.99 kg/m^2^; overweight: 25.00–29.99 kg/m^2^; and obesity: ≥30.00 kg/m^2^ [26]. Waist (WC) and hips circumferences were measured using measuring tape Seca 201 (Seca Gmbh & Co., Hamburg, Germany) to the nearest 0.1 cm. WC was measured at the end of several consecutive natural breaths, at a level parallel to the floor, midpoint between the top of the iliac crest and the lower margin of the last palpable rib in the midaxillary line. Hip circumference was measured at a level parallel to the floor, at the largest circumference of the buttocks. The following interpretation of WC was applied—increased cardiometabolic risk: ≥80 cm in females and ≥90 cm in males. Waist-to-height ratio (WtHR) was calculated, and the value ≥ 0.5 was considered as the cut-off point for increased cardiometabolic risk [27].

An Omron Body Fat Analyzer model HBF-360 (Omron Healthcare, Inc., Vernon Hills, IL, USA) was used to measure body fat. According to Malavolti et al. [28], this bioimpedance analysis is a valid, non-invasive, inexpensive method of determining total and regional body composition. The participants’ height, weight, age, and sex were entered into the analyzer. While standing with their feet slightly apart, the participant grasped the grip electrodes and held the analyzer in front of their body, with arms fully extended and parallel to the floor.

Physical activity (PA) level evaluation was carried out using the Polish version of the International Physical Activity Questionnaire (IPAQ)—Short Form [29] and performed according to the methodology described previously [5]. Metabolic equivalents were calculated and categorized accordingly: <600, 600–2999 and ≥3000 METs (min/week), and were classified as ‘*low*’, *moderate*’, or ‘*high*’ PA level, respectively.

Furthermore, to perform results analysis, the entire group of WLS users was additionally divided into tercile subgroups according to BM, percentage fat mass (FM), BMI, WC, hips circumference, WHR, and WtHR.

### 2.4. Review Procedures

Summarizing the methodological aspects and the flow of testing procedures, the survey questionnaire was designed to include all questions from the IPAQ (the Polish version of the validated tool); a dichotomous question (whether respondents use or not use WLS); and sociodemographic variables characterizing the respondents. During the anthropometric measurements, the researcher supervising the questionnaire asked the participants which specific dietary supplements they used. Once refined, the tool could be validated in further research. Questions regarding PA (in accordance with the methodology) addressed activities requiring PA in the last seven days. However, this is a short period and the question about WLS use required a longer time horizon. Thus, it was decided to adopt a timeframe of least three months when asking about WLS use. This time horizon could not be too long, as in declarative studies, respondents’ memory is crucial [30]. To mitigate memory suppression, a timeframe of three months was considered as optimal.

To interpret the associations between sociodemographic factors, health-related characteristics, and WLS use, the study was guided by the Social Determinants of Health model [31], which highlights how structural factors—such as education, income, and social position—influence access to and patterns of WLS use. In this model, behaviors and biological factors are considered as intermediary determinants of health. This conceptual model provides a concise multilevel lens for understanding variation in WLS use across demographic and health profiles.

### 2.5. Data Analysis

The examined variables were either nominal (sex, marital status, education, financial status, and use of types of WLS) or categorized (decade of life category, body mass and percentage fat mass terciles, BMI status/terciles, WC status/terciles, hips circumferences terciles, WtHR status/terciles, and PA level) and were presented by number and percentage distribution. The chi-square independence test was used to verify whether there were differences between the users of seven particular groups of WLS and each sociodemographic (sex, age, marital status, education, financial status) and anthropometric (body mass, BMI, WC, WtHR, hips circumference, WHR, BMI status, WHR status, percentage fat mass) measures, and PA level. One-way ANOVA was utilized to verify differences in body mass, BMI, WC, hips circumferences, WHR, WtHR, FM, and PA level between users of particular groups of WLS. All calculations were performed using Statistica 13.0 (StatSoft, Inc., Tulsa, OK, USA).

## 3. Results

The use of WLS was declared by 69.6% of participants (*n* = 745), and all further analyses were performed exclusively in this group. Females constitute 65.2% (*n* = 486) of WLS users. The mean age of the WLS consumers was 43.5 ± 14.9 years. Participants in their second decade of life constituted 1.3% of the analyzed group, third—22.4%, fourth—26.8%, fifth—16.8%, sixth—12.3%, seventh—17.9%, and eighth—2.4%. Participants being in a ‘relationship‘ constituted 56.0% of the analyzed group, while the remaining 44.0% indicated ‘no relationship’ status. According to education status, 3.2% participants had primary education, 34.4%—vocational, 33.2%—secondary, and 29.3% higher education. About 4.9% of participants described their financial status as ‘bad’, ‘hard to define*’*—26.0%, ‘good’—54.9%, and 14.1% as ‘very good’ (Table 1).

The most used WLS were aids *enhancing thermogenesis* (38.0% of respondents who were using WLS; Table 1), while less popular were the WLS *decreasing appetite* (15.0%), *blocking dietary fat absorption* (13.0%), *enhancing biochemical metabolism* (11.9%), *increasing water elimination* (10.1%), and *regulating metabolic rate* (8.1%). The least popular WLS were characterized by *complex/miscellaneous mechanisms* of action.

### 3.1. Sociodemographic Factors

Sex and age (decade of life) were the strongest determinants of the type of the WLS used (Table 1). Females, more frequently than males, used the following WLS: *enhancing water elimination* (97.3 vs. 2.7%), *regulating metabolic rate* (96.7 vs. 3.3%), *exhibiting complex/miscellaneous mechanisms of action* (89.7 vs. 10.3%), *blocking dietary fat absorption* (80.4 vs. 19.6%), and *decreasing appetite* (67.9 vs. 32.1%). Regarding decades of life, it was the greatest determinant of usage of the following WLS: *enhancing biochemical metabolism*, *regulating metabolic rate*, and *decreasing appetite*—the frequency of their usage was greater in the fifth-sixth and seventh-eight decades than in the second-third and fourth decades of life; *enhancing thermogenesis*—the lowest percentage of their users was noted in the seventh-eight decades (6.4% comparing to 36.4% in the second-third decades). Regarding marital status, the greatest discrepancy was observed for *WLS enhancing biochemical metabolism* (71.9 vs. 28.1%) and decreasing appetite (63.4 vs. 36.6%), which were more frequently used by respondents being ‘in a relationship’ (comparing to ‘no relationship’). Referring to education, regardless of the type of WLS, the lowest rates of their usage were noted among participants with primary education; still, the group was underrepresented compared to the remaining education groups. Among users of WLS possessing a *complex mechanism of action* and *increasing water elimination*, there was a notably greater share of respondents with higher (58.6 and 40.0%, respectively) education compared to secondary (20.7 and 24.0%) and vocational (20.7 and 29.3%) education, while among users of WLS *enhancing biochemical metabolism*, *regulating metabolic rate*, and *decreasing appetite*, there was a greater proportion of respondents with vocational (50.6, 46.7 and 41.1%, respectively) and secondary (28.1, 31.7 and 35.7%) education compared to individuals with higher education (16.9, 13.3 and 16.9%). Referring to the financial status, there was the greatest representation of respondents characterized by ‘good’ financial status among users of all analyzed types of WLS; they constitute about fifty percent of the users of each WLS group.

### 3.2. Anthropometrics, Body Fat and Physical Activity

When analyzing terciles of BM within users of particular WLS, it was noticed that for WLS *regulating metabolic rate* and *enhancing biochemical metabolism*, the proportion of participants from the third BM tercile (56.7 and 48.3% of users, respectively; Table 2) was relatively higher than participants from the first (15.0 and 29.2%) and the second (28.3 and 22.5%) BM terciles, while for supplements with *complex/miscellaneous mechanisms*, there was a greater proportion of respondents from the second (41.4% of users) and the first (37.9%) BM terciles compared to the share of individuals from the third BM tercile (20.7%). When analyzing the mean values of BM, the highest value was observed among individuals using WLS *regulating metabolic rate* (77.6 ± 2.2 kg), and the lowest among users of WLS with *complex/miscellaneous mechanisms of action* (69.3 ± 10.7 kg).

Surprisingly, among the users of WLS *enhancing thermogenesis*, the share of individuals from the first, second, and the third (51.2% of users) FM terciles was 51.2, 28.3, and 20.5%, respectively (Table 2). Among respondents ingesting WLS *regulating metabolic rate*, 71.7% were individuals from the third FM tercile, while 28.3 and 15.0% were individuals from the second and first terciles, respectively. The distributions of respondents, according to FM terciles, exhibited a similar pattern within the remaining WLS types (Table 2). The highest mean FM percentage was seen in consumers of WLS *regulating metabolic rate* (37.2 ± 9.4%), and the lowest in individuals ingesting WLS *enhancing thermogenesis* (26.9 ± 10.1%) (Table 3).

Based on the BMI classification, only about 50% of respondents who reported ingesting WLS were overweight or obese (Table 2). There was a great variability in the distribution of BMI status between types of WLS. Namely, the highest percentage of overweight and obese individuals (83.3%) was observed among consumers of WLS *regulating metabolic rate*, while the lowest was observed among respondents ingesting supplements *enhancing thermogenesis* (34.6%) and supplements possessing *complex/miscellaneous mechanisms of action* (37.9%; Table 2). The highest mean BMI was observed among consumers of WLS *regulating metabolic rate* (28.2 ± 4.4 kg/m^2^) and the lowest was observed among respondents ingesting supplements *enhancing thermogenesis* (23.7 ± 4.2 kg/m^2^; Table 3).

Based on WC (M ≥ 90 cm, F ≥ 80 cm), merely 24% of respondents ingesting WLS could be categorized as being at high health risk (Table 2). The highest mean WC was observed among consumers of WLS *enhancing biochemical metabolism* (82.0 ± 9.2 cm) and *regulating metabolic rate* (80.2 ± 10.4 cm) and the lowest was observed among respondents ingesting supplements *increasing water elimination* (75.8 ± 8.8 cm; Table 3).

Based on WtHR (≥0.05), about 23% of respondents using WLS could be categorized as being at high risk of cardiometabolic disorders (Table 2). The greatest percentage of individuals with WtHR ≥ 0.05 was observed among users of WLS *regulating metabolic rate* (41.7% of users), followed by WLS *enhancing biochemical metabolism* (34.8%), while the lowest was observed among users of WLS *enhancing thermogenesis* (15.5%). The highest mean WtHR was observed in consumers of WLS *regulating metabolic rate* (0.48 ± 0.06) and *enhancing biochemical metabolism* (0.47 ± 0.06), and the lowest was observed among consumers of supplements *enhancing thermogenesis* (0.44 ± 0.05; Table 3).

Based on the METs equivalents, 27% of WLS consumers were categorized as having low PA level, while 41 and 32% as having moderate and high PA levels, respectively (Table 2). Interestingly, among consumers of WLS *enhancing thermogenesis*, 18.3% had low PA level (the lowest contribution compared to the remaining WLS groups), and simultaneously, the percentage of people with excessive BMI (34.6%) in this particular group was also the lowest (Table 2). The mean MET was the highest in individuals reporting ingestion of WLS *enhancing thermogenesis* (2669 ± 1992 MET min/week)—it was significantly higher compared to values reported among consumers of WLS *regulating metabolic rate* (1625 ± 1785 MET min/week), *decreasing appetite* (1728 ± 1827 MET min/week), and *blocking dietary fat absorption* (2026 ± 2068 MET min/week).

## 4. Discussion

### 4.1. The Mismatch Between WLS Use and Clinical Indications

Among individuals who reported WLS use, only about fifty percent were overweight or obese according to BMI classification; 24% were at high health risk based on WC, and 23% were at high cardiometabolic risk based on WtHR. Thus, is seems that study participants should more carefully consider the necessity of WLS ingestion, especially in light of the possible risks related to uncontrolled WLS intake and their unclear effectiveness.

A recent systematic review by Batsis et al. [32] clearly demonstrated a low number of randomized controlled trials (RCT) providing high-quality evidence on the efficacy of particular dietary supplements for weight loss. Out of 315 RCTs included in the review, evaluating the effectiveness of fourteen different dietary supplements, alternative therapies, or a combination of both, only 16.5% studies (52 studies) were characterized by low risk of bias (RoB). Of these studies, only 31% (16 studies) indicated significant pre/post-intergroup differences in BM. Thus, the actual justification of WLS use remains unclear.

### 4.2. Thermogenesis Enhancers

In the current study, the most popular WLS were preparations aimed at *enhancing thermogenesis* (including L-carnitine, hydroxycitric acid, green tea, caffeine, licorice, bitter orange, guarana, yerba mate, conjugated linoleic acid). Nearly 40% of WLS consumers reported their intake. Consumers of supplements *enhancing thermogenesis* were significantly younger (37.2 ± 12.2 years) compared to consumers of WLS aimed at *enhancing biochemical metabolism* (55.1 ± 13.6 years), *regulating metabolic rate* (52.0 ± 15.1 years), *decreasing appetite* (48.7 ± 14.8 years), and *blocking dietary fat absorption* (43.8 ± 15.0 years). Interestingly, among consumers of the discussed class of WLS, mean WtHR was the lowest (0.44 ± 0.05) and significantly lower compared to the mean WtHR of consumers of WLS aimed at *regulating metabolic rate* (0.48 ± 0.06), *enhancing biochemical metabolism* (0.47 ± 0.06), or *decreasing appetite* (0.46 ± 0.05). Accordingly, individuals ingesting WLS *enhancing thermogenesis* were characterized by lower BM and lower percentage of FM and WC compared to respondents declaring intake of most of the other WLS. Yet, the number of METs was the highest in this group (2669 ± 1992 MET min/week) and significantly higher compared to users of WLS aimed at *regulating metabolic rate* (1625 ± 1785 MET min/week) and *decreasing appetite* (MET 1728 ± 1827 min/week).

The meta-analysis by Askarpour et al. [33], indicated that L-carnitine supplementation may have positive effects in supporting BM, BMI, and FM reduction, but not FM percentage or WC. Still, the effect was limited to overweight or obese individuals. However, there are some concerns related to L-carnitine safety. High doses of L-carnitine may contribute to increased concentration of trimethylamine-N-oxide (TMAO). TMAO can increase the risk of hypertension and atherosclerosis [34]. A parallel randomized placebo-controlled study in women aged 65–70 years revealed an increase of about ten times in TMAO concentration after 24 weeks of 1500 mg/day L-carnitine supplementation [35]. Still, the concentration of TMAO returned to the baseline value 4 months after cessation of supplementation and remained unchanged in the next 8 months. Risk analysis must be considered when considering L-carnitine supplementation. Hydroxycitric acid (HCA) is a compound of the *Garcinia cambogia* plant. The meta-analysis of 12 RCTs by Onakpoya et al. [36] indicated that HCA supplementation has a small, but statistically significant effect over placebo comparators on BM reduction. The more recent meta-analysis by Golzarand et al. [37] of eight RCTs indicated that the *Garcinia cambogia* supplement significantly reduced BM (~−1.34 kg), BMI (~−0.99 kg/m^2^), fat mass (~−0.42%), and WC (~−4.16 cm) compared with the placebo comparators. According to the newest literature [32], most of the RCTs examining the effect of *Garcinia cambogia* on BM reduction provide rather low-quality evidence. Although supplementation with *Garcinia cambogia*/HCA may lead to small/moderate reduction in BM, the clinical relevance of those changes are questionable. Moreover, cases of acute hepatitis after *Garcinia cambogia* extract supplementation have also been reported [38].

In addition, green tea extracts have been widely studied in improving the BM reduction process. Meta-analysis by Asbaghi [39] indicated that green tea extract supplementation may contribute to significant reduction in BM (mean difference −0.64 kg), FM percentage (~−0.63%), BMI (~−0.17 kg/m^2^), and malonyl dialdehyde concentration (~−0.33 μmol∙L^−1^) as well as an increase in adiponectin (~0.62 μg∙mL^−1^) and total antioxidant capacity of plasma (~0.11 mmol∙mL^−1^), with no effect of FM (in kg) or concentration of leptin and ghrelin compared to controls. Another meta-analysis of RCTs by Zhang et al. [40] revealed that green tea supplementation contributed to reduced BM (mean difference −1.23 kg), BMI (~−0.47  kg/m^2^), and WC (~−3.46 cm) in overweight and obese women. Based on the results of the dose–response assessment, it was concluded that healthcare professionals can recommend using green tea extract supplementation with dosages ≥ 1000 mg/day and duration ≥ 8 weeks in obese women. Still, according to the European Food Safety Authority opinion [41], (-)-epigallocatechin-3-gallate (EGCG—the most relevant catechin in green tea) dose ≥ 800 mg/day taken as a food supplement has been shown to induce a statistically significant increase in serum transaminases in treated subjects compared to controls. Having this in mind, the supplemented doses should be verified in respect of daily EGCG intake. Supplementation with another thermogenic agent, caffeine, has been indicated in the recent meta-analysis [42] as promoting reduction in BM, BMI, and FM.

According to the meta-analysis by Koncz et al. [43], bitter orange (*Citrus aurantium*) extracts and *p*-synephrine supplementation is not effective in supporting BM reduction or an improvement in body composition and it tends to raise blood pressure and heart rate. Moreover, the authenticity and safety of dietary supplements containing bitter orange extracts are questionable [44]. Licorice (*Glycyrrhiza glabra* L.) extract supplementation, although effective in evoking statistically significant (thought clinically not relevant) reduction in BM and BMI, was also shown to increase diastolic blood pressure [45]. A meta-analysis of 15 RCTs by Onakpoya et al. [46] revealed a significant difference in BM (mean difference −0.70 kg) and FM (~−1.33 kg) loss favoring conjugated linoleic acid over placebo. Yet, the magnitude of these effects was small, and the clinical relevance was uncertain.

### 4.3. Appetite Suppressants

The second-most popular group of WLS in the current study was aimed at *decreasing appetite* (including guar gum, pineapple, glucomannan, psyllium, and other fibers). Their ingestion was reported by 15.0% of participants declaring use of WLS. Being a female and being ‘in a relationship’ (in contrary to ‘no relationship’ status) favored the use of those supplements. Mean age of the group using WLS *decreasing appetite* (48.7 ± 14.8 yrs) was among the highest—it was lower solely compared to the users of supplements *enhancing biochemical metabolism*.

Regarding the efficacy of psyllium supplementation, the results of meta-analyses published so far are inconclusive [47,48]. A recent meta-analysis by Gibb et al. [47] of six studies with supplementation duration of at least 2 months and daily dose of psyllium at least 7 g, revealed a significant effect on reduction in BM (mean difference −2.1 kg), BMI (~−0.8  kg/m^2^), and WC (~−2.2 cm) in intervention groups compared to controls. In contrary, according to the meta-analysis by Mofrad et al. [48] of 22 RCTs, there was no significant effect of psyllium supplementation on BM. Similarly, the meta-analyses by Alaeian et al. [49] and Onakpoya et al. [50] revealed no effect of guar gum or glucomannan supplementation, respectively, in supporting BM reduction. There is solely one study on humans [51] investigating the effect of bromelain supplementation on BM. The results of the study suggest that 8-week bromelain supplementation in obese diabetic patients may contribute to improved obesity treatment. Still, no exact data on BM is given in the paper. Thus, the results of the study should be interpreted carefully.

### 4.4. Dietary Fat Absorption Blockers

About 13% of WLS consumers indicated the ingestion of agents *blocking dietary fat absorption* (i.e., chitosan). Being female was a strong determinant of using this type of WLS. The lowest mean WC (77.3 ± 10.0 cm) was observed among users of WLS *blocking dietary fat absorption*—but still, the 97.3% of users were females.

Rendering to the recent meta-analysis by Kholdebarin et al. [52], chitosan supplementation significantly reduced BM (mean difference −0.79 kg), percentage fat mass (~−0.41%), and increased fat-free mass (~0.20 kg), but had no significant impact on BMI and WC. Interestingly, an earlier paper by Mhurchu et al. [53] indicated that analyses involving all the extracted search studies via the literature revealed small but statistically significant and greater reduction in BM (mean difference −1.7 kg) in chitosan groups compared with placebo controls. Nevertheless, the analyses restricted to high-quality studies showed that reductions in body mass (~−0.6 kg) were less than in lower-quality studies (~−2.3 kg).

Supplements aiming at *increasing water elimination* were ingested by 10.1% of WLS users. However, there is no scientific background to support their usage in BM reduction. Among the adverse effects of their use are dehydration and electrolyte imbalance [54].

### 4.5. Strengths and Limitations

Finally, the undeniable strength of the current study refers to the fact that all the questionnaires and measurements were performed during the face-to-face interviews. The interviewers were trained to perform all the measurements in a standardized manner. They also had a degree in Pharmacy and hence were able to correctly categorize WLS. Moreover, this is the first study to investigate the usage of the broad range of types of WLS.

This study has several limitations related to the scope of available data. Important factors commonly considered in studies of weight loss-related outcomes—such as dietary intake or energy deficit, individual motivation for weight loss, previous weight loss attempts, detailed comorbidity profiles, medication use (including drugs with potential interactions with WLS), smoking status, and alcohol consumption—were not collected and therefore could not be analyzed. The absence of these variables may limit the interpretation of the results and restrict the ability to control for potential confounders. Future studies should include these clinically relevant and behavioral factors to provide a more comprehensive understanding of the mechanisms and determinants of WLS use among adult Poles. Moreover, the current study did not consider the amount of active compounds, daily intake, poly-supplement use, or duration of supplementation (determination of WLS consumed in the last three months was the main objective). Including these data would considerably strengthen the results and should be incorporated in the future investigations. What is more, the implemented procedure of gathering data regarding WLS was newly developed and has not been adapted or validated in previously published studies. Still, the face-to-face review method seems to justify the appropriateness of the implemented methodology. The convenience sampling method and cross-sectional nature should be taken into account when interpreting the results of the current study.

## 5. Conclusions

The results of this cross-sectional study indicated high prevalence of WLS intake and their common ingestion by individuals characterized by normal BM, based on BMI classification (43.4% of WLS users had normal BM and 6.3% were underweight), or by individuals with no increased cardiometabolic risk based on WC (76.1% of WLS users) or WtHR (76.6%). The most popular WLS were supplements aiming at *enhancing thermogenesis*—they were equally popular among females and males. Based on the previous literature, the efficiency and safety of various compounds advertised as thermogenesis enhancers is limited. Females, more frequently than males, were using supplements aimed at *regulating metabolic rate* and *increasing water elimination*. Interestingly, among consumers of supplements aiming at *enhancing thermogenesis*, the highest mean MET was observed; simultaneously, the contribution of overweight/obese individuals was the lowest in this group (comparing to the consumers of the remaining WLS). This observation would align with the results of the recent systematic review and meta-analysis, indicating the superior effectiveness of thermogenic agents combined with physical activity as a strategy of body fat management (compared to thermogenic aids alone) [55]. Extensive educational programs are essential for society regarding rational, effective, and safe methods of reducing excessive BM, taking into consideration sociodemographic factors, anthropometric parameters, and physical activity of the WLS consumers. In addition to education, strengthening regulatory oversight and monitoring of the dietary supplement market may further help reduce inappropriate WLS use and promote informed, evidence-based decisions related to body mass management. The implementation of corporate social responsibility among WLS market suppliers would also be desirable.

## Figures and Tables

**Table 1:** Sociodemographic factors and the use of weight loss supplements.

Sociodemographic Factors	Weight Loss Supplements							
	Enhancing Thermogenesis	Decreasing Appetite	Blocking Dietary Fat Absorption	Increasing Water Elimination	Enhancing Biochemical Metabolism	Regulating Metabolic Rate	Complex/Miscellaneous Mechanisms	*p*-Value
								*χ* ^2^
	*n* (%)	*n* (%)	*n* (%)	*n* (%)	*n* (%)	*n* (%)	*n* (%)	
**All (*n* = 745)**	283 (38.0)	112 (15.0)	97 (13.0)	75 (10.1)	89 (11.9)	60 (8.1)	29 (3.9)	
**Sex**								
Males (*n* = 259)	153 (54.1)	36 (32.1)	19 (19.6)	2 (2.7)	44 (49.4)	2 (3.3)	3 (10.3)	<0.001
Females (*n* = 486)	130 (45.9)	76 (67.9)	78 (80.4)	73 (97.3)	45 (50.6)	58 (96.7)	26 (89.7)	132.9
**Decade of life**								
Second-third (*n* = 177)	103 (36.4)	16 (14.3)	19 (19.6)	19 (25.3)	8 (9.0)	7 (11.7)	5 (17.2)	
Fourth (*n* = 200)	82 (29.0)	24 (21.4)	37 (38.1)	27 (36.0)	8 (9.0)	9 (15.0)	13 (44.8)	<0.001
Fifth-sixth (*n* = 217)	80 (28.3)	38 (33.9)	18 (18.6)	20 (26.7)	30 (33.7)	21 (35.0)	10 (34.5)	152.7
Seventh-eighth (*n* = 151)	18 (6.4)	34 (30.4)	23 (23.7)	9 (12.0)	43 (48.3)	23 (38.3)	1 (3.4)	
**Marital status**								
No relationship (*n* = 328)	154 (54.4)	41 (36.6)	41 (42.3)	31 (41.3)	25 (28.1)	24 (40.0)	12 (41.4)	<0.001
In relationship (*n* = 417)	129 (45.6)	71 (63.4)	56 (57.7)	44 (58.7)	64 (71.9)	36 (60.0)	17 (58.6)	24.9
**Education**								
Higher (*n* = 218)	89 (31.4)	22 (19.6)	37 (38.1)	30 (40.0)	15 (16.9)	8 (13.3)	17 (58.6)	
Secondary (*n* = 247)	110 (38.9)	40 (35.7)	29 (29.9)	18 (24.0)	25 (28.1)	19 (31.7)	6 (20.7)	<0.001
Vocational (*n* = 256)	81 (28.6)	46 (41.1)	28 (28.9)	22 (29.3)	45 (50.6)	28 (46.7)	6 (20.7)	65.1
Primary (*n* = 24)	3 (1.1)	4 (3.6)	3 (3.1)	5 (6.7)	4 (4.5)	5 (8.3)	0 (0.0)	
**Financial status**								
Very good (*n* = 105)	50 (17.7)	11 (9.8)	15 (15.5)	12 (16.0)	3 (3.4)	3 (5.0)	11 (37.9)	
Good (*n* = 409)	169 (59.7)	61 (54.5)	50 (51.5)	38 (50.7)	51 (57.3)	27 (45.0)	13 (44.8)	<0.001
Hard to define (*n* = 194)	58 (20.5)	34 (30.4)	29 (29.9)	20 (26.7)	29 (32.6)	21 (35.0)	3 (10.3)	59.6
Bad (*n* = 37)	6 (2.1)	6 (5.4)	3 (3.1)	5 (6.7)	6 (6.7)	9 (15.0)	2 (6.9)	

Note: The data were analyzed with the chi-square independence test.

**Table 2:** Use of weight loss supplements according to anthropometric factors and physical activity of participants.

Indicator	Weight Loss Supplements							
	Enhancing Thermogenesis	Decreasing Appetite	Blocking Dietary Fat Absorption	Increasing Water Elimination	Enhancing Biochemical Metabolism	Regulating Metabolic Rate	Complex/Miscellaneous Mechanisms	*p*-Value
								*χ* ^2^
	*n*(%)	*n*(%)	*n*(%)	*n*(%)	*n*(%)	*n*(%)	*n*(%)	
**All (*n* = 745)**	283 (38.0)	112 (15.0)	97 (13.0)	75 (10.1)	89 (11.9)	60 (8.1)	29 (3.9)	
**Body mass tercile**								
First tercile (*n* = 226)	94 (33.2)	32 (28.6)	28 (28.9)	26 (34.7)	26 (29.2)	9 (15.0)	11 (37.9)	<0.001
Second tercile (*n* = 259)	113 (39.9)	37 (33.0)	32 (33.0)	28 (37.3)	20 (22.5)	17 (28.3)	12 (41.4)	35.5
Third tercile (*n* = 260)	76 (26.9)	43 (38.4)	37 (38.1)	21 (28.0)	43 (48.3)	34 (56.7)	6 (20.7)	
**Fat mass tercile**								
First tercile (*n* = 261)	145 (51.2)	33 (29.5)	27 (27.8)	16 (21.3)	27 (30.3)	6 (10.0)	7 (24.1)	<0.001
Second tercile (*n* = 221)	80 (28.3)	34 (30.4)	29 (29.9)	28 (37.3)	26 (29.2)	11 (18.3)	13 (44.8)	90.5
Third tercile (*n* = 263)	58 (20.5)	45 (40.2)	41 (42.3)	31 (41.3)	36 (40.4)	43 (71.7)	9 (31.0)	
**BMI tercile**								
First tercile (*n* = 247)	114 (40.3)	31 (27.7)	34 (35.1)	23 (30.7)	25 (28.1)	9 (15.0)	11 (37.9)	<0.001
Second tercile (*n* = 240)	104 (36.7)	35 (31.3)	28 (28.9)	29 (38.7)	22 (24.7)	11 (18.3)	11 (37.9)	56.6
Third tercile (*n* = 258)	65 (23.0)	46 (41.1)	35 (36.1)	23 (30.7)	42 (47.2)	40 (66.7)	7 (24.1)	
**BMI status**								
Underweight (*n* = 47)	20 (7.1)	6 (5.4)	7 (7.2)	1 (1.3)	7 (7.9)	3 (5.0)	3 (10.3)	<0.001
Normal (*n* = 323)	165 (58.3)	38 (33.9)	33 (44.0)	33 (44.0)	32 (36.0)	7 (11.7)	15 (51.7)	88.1
Overweight (*n* = 243)	69 (24.4)	49 (43.8)	35 (36.1)	28 (37.3)	31 (34.8)	23 (38.3)	8 (27.6)	
Obese (*n* = 132)	29 (10.2)	19 (17.0)	22 (22.7)	13 (17.3)	19 (21.3)	27 (45.0)	3 (10.3)	
**Waist circumference tercile**								
First tercile (*n* = 231)	98 (34.6)	34 (30.4)	32 (33.0)	25 (33.3)	17 (19.1)	15 (25.0)	10 (34.5)	<0.001
Second tercile (*n* = 245)	110 (38.9)	31 (27.7)	29 (29.9)	26 (34.7)	29 (32.6)	11 (18.3)	9 (31.0)	33.9
Third tercile (*n* = 269)	75 (26.5)	47 (42.0)	36 (37.1)	24 (32.0)	43 (48.3)	34 (56.7)	10 (34.5)	
**Waist circumference**								
M < 90 cm, F < 80 cm (*n* = 567)	238 (84.1)	85 (75.9)	73 (75.3)	56 (74.7)	63 (70.8)	31 (51.7)	21 (72.4)	<0.001
M ≥ 90 cm, F ≥ 80 cm (*n* = 178)	45 (15.9)	27 (24.1)	24 (24.7)	19 (25.3)	26 (29.2)	29 (48.3)	8 (27.6)	31.4
**Hips circumference tercile**								
First tercile (*n* = 235)	100 (35.3)	31 (27.7)	26 (26.8)	28 (37.3)	22 (24.7)	19 (31.7)	9 (31.0)	
Second tercile (*n* = 241)	105 (37.1)	36 (32.1)	34 (35.1)	20 (26.7)	23 (25.8)	15 (25.0)	8 (27.6)	0.04
Third tercile (*n* = 269)	78 (27.6)	45 (40.2)	37 (38.1)	27 (36.0)	44 (49.4)	26 (43.3)	12 (41.4)	21.8
**WHR tercile**								
First tercile (*n* = 240)	98 (34.6)	39 (34.8)	39 (40.2)	23 (30.7)	17 (19.1)	16 (26.7)	8 (27.6)	
Second tercile (*n* = 252)	98 (34.6)	35 (31.3)	32 (33.0)	29 (38.7)	33 (37.1)	14 (23.3)	11 (37.9)	0.05
Third tercile (*n* = 253)	87 (30.7)	38 (33.9)	26 (26.8)	23 (30.7)	39 (43.8)	30 (50.0)	10 (34.5)	21.1
**WtHR tercile**								
First tercile (*n* = 247)	106 (37.5)	36 (32.1)	33 (34.0)	29 (38.7)	20 (22.5)	12 (20.0)	11 (37.9)	
Second tercile (*n* = 244)	107 (37.8)	28 (25.0)	32 (33.0)	25 (33.3)	26 (29.2)	16 (26.7)	10 (34.5)	<0.001
Third tercile (*n* = 254)	70 (24.7)	48 (42.9)	32 (33.0)	21 (28.0)	43 (48.3)	32 (53.3)	8 (27.6)	36.8
**WtHR**								
<0.05 (*n* = 571)	239 (84.5)	80 (71.4)	76 (78.4)	60 (80.0)	58 (65.2)	35 (58.3)	23 (79.3)	
≥0.05 (*n* = 174)	44 (15.5)	32 (28.6)	21 (21.6)	15 (20.0)	31 (34.8)	25 (41.7)	6 (20.7)	<0.001
**Physical activity level**								29.9
Low (*n* = 153)	40 (18.3)	31 (37.3)	23 (32.9)	15 (28.3)	22 (30.6)	17 (39.5)	5 (20.8)	
Moderate (*n* = 229)	92 (40.2)	32 (38.6)	26 (37.1)	23 (43.4)	29 (40.3)	18 (41.9)	9 (37.5)	0.025
High (*n* = 181)	86 (39.4)	20 (24.1)	21 (30.0)	15 (28.3)	21 (29.2)	8 (18.6)	10 (41.7)	23.4

Note: The data were analyzed with the chi-square independence test.

**Table 3:** Age and anthropometric and physical activity factors across consumers of weight loss supplements.

Indicator	Weight Loss Supplements							
	Enhancing Thermogenesis	Decreasing Appetite	Blocking Dietary Fat Absorption	Increasing Water Elimination	Enhancing Biochemical Metabolism	Regulating Metabolic Rate	Complex/Miscellaneous Mechanisms	*p*-Value
								*F*
**Age (years)**								
All	37.2 ± 12.2 ^a^	48.7 ± 14.8 ^c^	43.8 ± 15.0 ^b^	39.9 ± 12.6 ^ab^	55.1 ± 13.6 ^d^	52.0 ± 15.1 ^cd^	38.9 ± 10.7 ^ab^	<0.000; 29.9
Males	35.5 ± 9.9 ^c^	54.1 ± 13.5 ^b^	48.6 ± 17.4 ^ab^	48.0 ± 28.3 ^abcd^	59.9 ± 9.2 ^d^	53.0 ± 0.0 ^abd^	37.3 ± 6.4 ^ac^	<0.000; 35.6
Females	39.2 ± 14.2 ^a^	46.1 ± 14.8 ^bc^	42.7 ± 14.2 ^ab^	39.7 ± 12.2 ^a^	50.4 ± 15.5 ^cd^	52.0 ± 15.4 ^d^	39.1 ± 11.1 ^a^	<0.000; 9.05
**Body mass (kg)**								
All	72.0 ± 11.8 ^a^	73.5 ± 12.7 ^ab^	73.1 ± 12.9 ^ab^	71.4 ± 10.6 ^a^	76.2 ± 13.7 ^bc^	77.6 ± 12.2 ^c^	69.3 ± 10.7 ^a^	0.002; 3.45
Males	73.7 ± 10.3 ^b^	79.2 ± 12.3 ^a^	78.5 ± 12.9 ^ab^	67.0 ± 12.7 ^ab^	77.7 ± 13.3 ^a^	89.0 ± 8.5 ^ab^	69.8 ± 7.0 ^ab^	0.020; 2.56
Females	70.0 ± 13.1 ^a^	70.8 ± 12.1 ^ab^	71.7 ± 12.6 ^ab^	71.5 ± 10.6 ^ab^	74.8 ± 14.2 ^bc^	77.2 ± 12.2 ^c^	69.2 ± 11.1 ^ab^	0.006; 3.03
**Fat mass (%)**								
All	26.9 ± 10.1 ^a^	32.1 ± 9.7 ^a^	31.7 ± 10.1 ^a^	32.5 ± 9.1 ^a^	32.0 ± 10.8 ^a^	37.2 ± 9.4 ^c^	28.9 ± 10.2 ^ab^	<0.000; 12.38
Males	24.3 ± 8.5 ^a^	33.1 ± 9.4 ^b^	31.9 ± 8.9 ^b^	27.0 ± 16.9 ^ab^	31.8 ± 10.1 ^b^	38.1 ± 3.1 ^b^	20.2 ± 10.9 ^a^	<0.000; 8.75
Females	29.9 ± 10.9 ^a^	31.6 ± 9.9 ^a^	31.6 ± 10.4 ^a^	32.7 ± 9.0 ^a^	32.3 ± 11.5 ^a^	37.1 ± 9.5 ^b^	29.9 ± 9.9 ^a^	0.002; 3.61
**BMI (kg/m^2^)**								
All	23.7 ± 4.2 ^b^	25.6 ± 4.3 ^a^	25.5 ± 4.7 ^a^	25.5 ± 4.0 ^a^	25.5 ± 4.9 ^a^	28.2 ± 4.4 ^c^	24.1 ± 4.3 ^ab^	<0.000; 10.43
Males	23.0 ± 3.5 ^a^	26.5 ± 4.4 ^c^	26.3 ± 4.6 ^c^	23.8 ± 6.5 ^abc^	25.4 ± 4.6 ^a^	29.1 ± 1.3 ^a^	21.4 ± 3.5 ^ab^	<0.000; 6.69
Females	24.6 ± 4.8 ^a^	25.2 ± 4.3 ^a^	25.3 ± 4.8 ^a^	25.5 ± 4.0 ^a^	25.6 ± 5.2 ^a^	28.2 ± 4.5 ^b^	24.4 ± 4.3 ^a^	<0.000; 4.50
**Waist circumference (cm)**								
All	77.5 ± 8.1 ^a^	78.2 ± 9.0 ^ab^	77.3 ± 10.0 ^a^	75.8 ± 8.8 ^a^	82.0 ± 9.2 ^c^	80.2 ± 10.4 ^bc^	77.5 ± 9.5 ^ab^	<0.000; 4.53
Males	78.7 ± 7.1 ^b^	82.8 ± 8.6 ^ac^	82.8 ± 8.8 ^ac^	76.8 ± 8.1 ^ab^	82.5 ± 8.2 ^ac^	93.0 ± 12.7 ^c^	75.3 ± 5.1 ^ab^	0.001; 3.80
Females	76.0 ± 8.9 ^a^	76.1 ± 8.4 ^a^	75.9 ± 9.9 ^a^	75.8 ± 8.9 ^a^	81.5 ± 10.2 ^b^	79.8 ± 10.1 ^b^	77.7 ± 10.0 ^ab^	0.003; 3.40
**Hips circumference (cm)**								
All	95.5 ± 8.0 ^b^	98.0 ± 7.7 ^a^	98.6 ± 8.8 ^a^	97.6 ± 7.9 ^ab^	99.9 ± 9.4 ^a^	99.5 ± 9.0 ^a^	97.5 ± 7.9 ^ab^	<0.000; 4.95
Males	93.3 ± 7.6 ^b^	97.9 ± 7.8 ^ac^	99.7 ± 11.2 ^a^	92.0 ± 9.9 ^abc^	98.5 ± 10.0 ^a^	99.5 ± 2.1 ^abc^	88.5 ± 7.0 ^bc^	<0.001; 4.58
Females	98.1 ± 7.7	98.1 ± 7.8	98.4 ± 8.1	97.7 ± 7.8	101.3 ± 8.7	99.5 ± 9.2	98.5 ± 7.4	0.289; 1.23
**WHR**								
All	0.81 ± 0.06 ^de^	0.80 ± 0.07 ^bc^	0.78 ± 0.06 ^ab^	0.78 ± 0.06 ^a^	0.82 ± 0.05 ^e^	0.81 ± 0.07 ^cde^	0.79 ± 0.06 ^abcd^	<0.000; 6.42
Males	0.84 ± 0.04 ^a^	0.85 ± 0.06 ^a^	0.83 ± 0.02 ^a^	0.83 ± 0.00 ^a^	0.84 ± 0.03 ^a^	0.94 ± 0.15 ^b^	0.85 ± 0.02 ^a^	0.039; 2.25
Females	0.77 ± 0.06 ^a^	0.78 ± 0.06 ^a^	0.77 ± 0.06 ^a^	0.77 ± 0.06 ^a^	0.80 ± 0.06 ^b^	0.80 ± 0.07 ^b^	0.79 ± 0.06 ^ab^	0.005; 3.13
**WtHR**								
All	0.44 ± 0.05 ^a^	0.46 ± 0.05 ^bc^	0.46 ± 0.06 ^ab^	0.45 ± 0.05 ^ab^	0.47 ± 0.06 ^cd^	0.48 ± 0.06 ^d^	0.46 ± 0.07 ^abc^	<0.000; 7.10
Males	0.44 ± 0.05 ^a^	0.48 ± 0.05 ^c^	0.48 ± 0.05 ^c^	0.46 ± 0.07 ^abc^	0.47 ± 0.05 ^c^	0.53 ± 0.06 ^c^	0.42 ± 0.05 ^ab^	<0.000; 6.36
Females	0.45 ± 0.05 ^a^	0.45 ± 0.05 ^a^	0.45 ± 0.05 ^a^	0.45 ± 0.05 ^a^	0.48 ± 0.06 ^b^	0.48 ± 0.06 ^b^	0.46 ± 0.07 ^ab^	<0.000; 3.84
**Physical activity level (MET min** **/** **week)**								
All	2669 ± 1992 ^b^	1728 ± 1827 ^a^	2026 ± 2068 ^a^	2127 ± 1885 ^ab^	2268 ± 2125 ^ab^	1625 ± 1785 ^a^	2411 ± 1737 ^ab^	0.002; 3.58
Males	2778 ± 1918 ^b^	1664 ± 1793 ^a^	2424 ± 1861 ^ab^	6584 ^c^	2549 ± 2102 ^ab^	1092 ± 1171 ^ab^	1515 ± 1104 ^ab^	0.036; 2.31
Females	2549 ± 2073	1759 ± 1858	1935 ± 2117	2042 ± 1796	2031 ± 2142	1651 ± 1815	2539 ± 1792	0.103; 1.78

Note: The results are expressed as the mean ± standard deviation. The data were analyzed with one-way ANOVA. ^a, b, c, d, e^ different letters refer to significant differences between groups of WLS.

## Data Availability

The original contributions presented in the study are included in the article; further inquiries can be directed at the corresponding author.

## Abbreviations

BM	Body mass
BMI	Body mass index
EGCG	(-)-epigallocatechin-3-gallate
HCA	Hydroxycitric acid
MET	Metabolic equivalent of a task
FM	Fat mass
PA	Physical activity
RCT	Randomized controlled trials
TMAO	Trimethylamine-N-oxide
WC	Waist circumference
WLS	Weight loss supplements
WHR	Waist-to-hips ratio
WtHR	Waist-to-height ratio
